# A signature-agnostic test for differences between tumor mutation spectra reveals carcinogen and ancestry effects

**DOI:** 10.1101/2025.05.15.654154

**Published:** 2025-05-19

**Authors:** Samuel F.M. Hart, Nicolas Alcala, Alison F. Feder, Kelley Harris

**Affiliations:** 1. Department of Genome Sciences, University of Washington, Seattle, USA; 2. Computational Cancer Genomics Team, International Agency for Research on Cancer (IARC/WHO), Genomic Epidemiology Branch, Lyon, France; 3. Herbold Computational Biology Program, Fred Hutch Cancer Center, Seattle, WA

## Abstract

Mutational signatures contain valuable information about the mutational processes shaping cancer genomes. However, despite dozens of tools to identify signatures in cancer samples, there is not an established metric for statistically comparing mutational signature results and quantifying the overall significance of differences among complex mixtures of signatures. To close this methodological gap, we demonstrate that a signature-agnostic metric for measuring differences in mutation spectra - the aggregate mutation spectrum distance permutation method (AMSD) - can discover differences overlooked by signature analysis. First, we reanalyzed a study of carcinogen exposure in mice, identifying statistically significant shifts in mutation spectra caused by eleven of twenty tested carcinogens. Only three carcinogens were previously reported to induce distinct mutation signatures, suggesting that many carcinogens perturb mutagenesis by altering the composition of endogenous signatures rather than introducing unique signatures. Next, we used human tumor data to determine whether patient ancestry has a measurable impact on tumor mutation spectra, finding significant ancestry-associated differences across ten cancer types: for example, Africans have elevated SBS4 in lung adenocarcinomas, East Asians have elevated SBS16 in esophageal and liver cancers plus elevated SBS10a/b in uterine and colorectal cancers, and Europeans have elevated SBS17b in esophageal cancers plus elevated SBS2/13 in bladder cancers. These examples suggest that AMSD is a robust tool for detecting differences among tumor mutation spectra, complementing signature-based approaches and enabling the discovery of environmental and genetic influences on mutagenesis in large datasets.

## Introduction

Mutation rates and biases in mutational sequence context can be influenced by endogenous cellular mechanisms, environmental exposures, and genetic variation. The relative frequency distribution of different possible mutation types in a genome, termed the mutation spectrum, encodes rich information about the processes that have shaped that genome’s mutational landscape, which can be especially complex in the context of cancers. To interpret this complexity, a framework has been introduced for decomposing the mutation spectra of thousands of tumors across dozens of cancer types to identify mutational signatures, characteristic mutation type distributions that are thought to reflect underlying mutational processes ^1–3^. Over 100 mutational signatures are currently listed in the COSMIC database (Catalogue Of Somatic Mutations In Cancer), with the majority being single base substitution (SBS) signatures, which look at the frequency of nucleotide changes in their 3mer flanking nucleotide context (e.g. CCG>CTG)^4^. It is now common practice when analyzing cancer genome sequencing data to infer what mutational signatures are active in a given sample, revealing key information about each individual cancer’s oncogenesis and evolutionary history.

While mutational signature analysis is effective for identifying *why* two mutation spectra may differ, it is not ideal for testing *whether or not* two spectra differ significantly from one another. Despite the existence of over 30 tools to identify signatures in cancer samples, there is not an established metric in signature fitting for statistically comparing signature fitting results to one another^5,6^. Mutational signature-based analyses can thus fall short of answering hypothesis-driven questions of interest, such as which carcinogens affect mutation spectra or whether there are differences in mutation spectra between populations. For example, a recent study by Riva, et al.^7^ identified carcinogens that cause distinct mutational signatures absent in unexposed tumors, but did not investigate the significance of variation in endogenous signature exposures. Additionally, signature fitting may not always reliably detect every mutational signature that is present in a dataset, due to issues such as low mutation counts, similar signatures, “flat” signatures, missing signatures, signature variability, signature overlap, or overfitting (Table 1). Although signatures can help with functional interpretation of mutation spectrum differences and the removal of mutations caused by technical artifacts, they do not always reduce the dimensionality of mutation spectrum data in a way that makes it easier to see patterns, as the number of annotated SBS signatures (86 in COSMIC v3.4) approaches the size of the 96-dimensional 3mer mutation state space. As cancer sequencing datasets grow larger and gain the resolution to test more hypotheses about what factors influence cancer mutations, the ability to detect significant differences between mutation spectra will be critical for making the most of the information content of these datasets.

Recently, Sasani, et al developed a metric called the aggregate mutation spectrum distance (AMSD) that measures the differences among the total mutation spectra of a set of samples^8^. This metric was instrumental for mapping genetic variants that affected germline mutagenesis in a recombinant inbred mouse panel due to its ability to detect relatively small perturbations caused by mutator loci. Although Kucab, et al. used a similar distance-based approach to identify carcinogens that significantly perturbed mutation spectra of cell lines^9^, analyses of tumor mutation spectra usually perform signature decomposition as a first step rather than directly analysing the signals present in the raw mutation spectra. Here, we show that AMSD is a powerful tool for testing hypotheses about what variables affect the complex mutation spectra of cancer genomes, in some cases resolving structure that is obscured by traditional signature analysis.

## Results

### AMSD is well-powered to detect quantitative differences in signature exposure using a permutation approach

**Figure 1.**
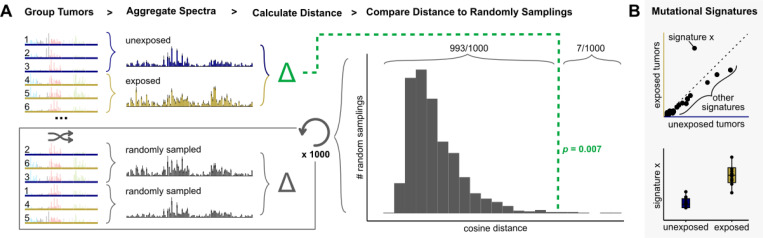
AMSD: a method to detect significant differences between mutation spectra. (**A**) Given mutation spectra for each sample in a cohort divided into two groups, such as tumors unexposed (blue) or exposed (yellow) to a carcinogen, AMSD aggregates mutation spectra for each group and calculates the cosine distance between the aggregate spectra (green). AMSD also randomly reshuffles group labels to calculate the cosine distance between randomly sampled tumors (grey), repeating 1000+ times to create a null distribution expectation. AMSD then calculates a *p*-value from the fraction of random samplings that are greater than or equal to the observed distance between the two groups, as visualized in the histogram. (**B**) To interpret what mutational mechanisms may underlie a significant difference, mutational signature fitting can be applied to the aggregate spectra (top) or individual samples (bottom) and compared for variables that AMSD finds have a significant effect on mutation spectra.

The aggregate mutation spectrum distance test (AMSD) is a permutation-based method for measuring the statistical significance of mutation spectrum differences between two groups, such as when comparing a carcinogen-exposed cohort to a control cohort (Figure 1A). AMSD quantifies the difference between the two spectra by calculating a distance metric (e.g. cosine distance), and then uses permutations to randomly reshuffle group labels across samples, recalculating the distance between the mutation spectra of thousands of randomly subsampled groups to generate a null distribution expectation, assuming no true differences between the groups. The fraction of permutations that generate a larger or equal distance than that observed between the real groups can be interpreted as the *p*-value significance of the difference between the two groups’ aggregate mutation spectra. Significant differences can then be examined using mutational-signature-based approaches to investigate drivers of mutational divergence (Figure 1B), which could be biological or artifactual.

Applying AMSD prior to mutational signature analysis has several advantages. Most notably, it provides an unbiased test for whether a statistically significant difference exists prior to interpreting that difference using signatures. In addition, it outputs a single comparison between the groups, reducing output dimensionality and minimizing the multiple testing burden that would be imposed by fitting signatures and then statistically comparing each pair of signature exposures. This is particularly useful when screening many variables for mutagenic effects, which can cause the multiple testing burden to quickly drown out the signal. One can then investigate potential drivers by comparing signature exposures only for variables that AMSD flags as significantly affecting spectra. Since the null permutations retain the data comparison structure, AMSD controls for unequal sample sizes and mutation counts between groups. The shape of the null mutation spectrum distance distribution can also be informative about the structure of the data – for example, a bimodal distribution indicates that spectrum subtypes or outliers are likely driving much of the variation in the data set. For more details on considerations when running AMSD, see methods.

To gain intuition about when AMSD is sufficiently powered to detect mutation spectrum differences, we applied the method to several simulated datasets. We tested AMSD’s ability to detect a difference between an exposure group and a control group while varying sample size (i.e., the number of tumors in each comparison group), mutation count, signature “flatness”, and signature exposure level. We observed that AMSD can reliably detect a signal when at least two of the following four parameters are met: sample size is large (hundreds), mutation count is high (WGS rather than WES), the variable signature is “spiky” (dominated by a small number of triplet contexts such as SBS2 rather than distributed more uniformly across contexts like SBS40), or relative exposure of the variable signature is high (>10% mutations/sample) (Supp Fig 1). Although real data sets are likely to be noisier and contain many confounding variables, these simulations provide a framework to interpret when AMSD might be powered to detect a signal and in which cases a negative result may reflect lack of power rather than true mutational homogeneity.

### Many carcinogens influence tumor mutation spectra via shifts in relative exposure to endogenous signatures

Environmental exposures to carcinogens such as ultraviolet light and smoking are responsible for some of the most striking and interpretable mutational signatures. However, most carcinogens lack such a striking characteristic signature and have not been reported to have a measurable effect on mutation load or mutational signatures in human cancer databases. To investigate carcinogens in a controlled setting, Riva et al. exposed mice to 20 different known or suspected carcinogens and then used whole genome sequencing to read out mutations from the resulting lung and liver tumors, extracting mouse mutational signatures *de novo* and comparing signature exposures between carcinogen-exposed tumors and spontaneously arising tumors in unexposed mice^7^. Surprisingly, they found that only three of these carcinogens significantly increased mutation load, and only three caused distinct mutational signatures: cobalt, vinylidene chloride (VDC), and 1,2,3-trichloropropane (TCP). However, we noted qualitative differences among the proportions of signature exposures in several other carcinogen-exposed tumors from this dataset. We hypothesized that some mutagens might increase the rates of certain endogenous mutational processes or interfere with their repair, and so we applied AMSD to test which mutagens were associated with significant shifts in the overall mutation spectrum composition.

**Figure 2.**
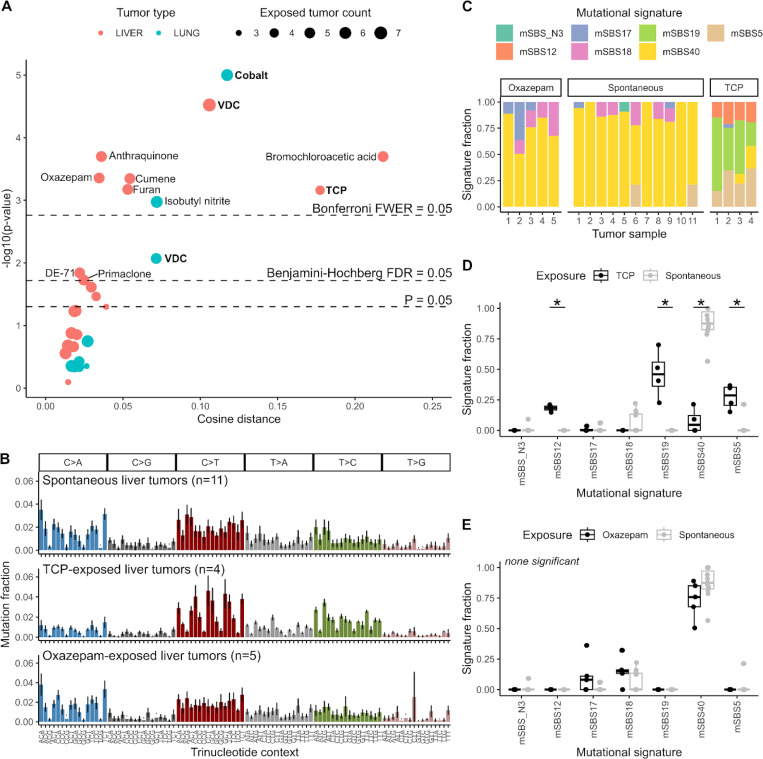
Many carcinogens affect mouse mutation spectra. (**A**) Volcano plot of AMSD results comparing carcinogen-exposed tumors to spontaneous tumors of the same tumor type (liver = red, lung = blue). Point sizes denote the number of carcinogen-exposed tumors, compared to *n* = 11 spontaneous liver tumors or *n* = 12 spontaneous lung tumors. Dashed lines correspond to significance thresholds: individual *P* = 0.05 threshold, Benjamini-Hochberg corrected false discovery rate (FDR) adjusted threshold, and Bonferroni corrected family-wise error rate (FWER) adjusted threshold. Carcinogen-exposed tumors in bold denote those with specific carcinogen-induced mutational signatures reported by Riva et al^7^. (**B**) Aggregate mean mutation spectra for spontaneous, TCP-exposed, and oxazepam-exposed liver tumors, with standard deviation in error bars. (**C**) Stacked bar plots of mutational signature exposure fractions for each sample in B. Mutational signatures were extracted de novo by Riva et al. (**D, E**) Side-by-side comparisons for each signature comparing TCP-exposed (**D**) or oxazepam-exposed (**E**) to spontaneous liver tumors. Asterisks denote *P* < 0.05 (both Wilcoxon rank sum test and two-tailed *t*-test with unequal variance were run and were in agreement in all cases).

Of 29 mutation spectrum comparisons (liver tumors exposed to one of 20 carcinogens versus spontaneous liver tumors, lung tumors exposed to one of 9 carcinogens versus spontaneous lung tumors), 15 revealed differences between exposed and unexposed tumors at a significance level of *p* < 0.05. 12 of these remained significant after Benjamini-Hochberg correction for multiple testing, and 9 remained significant after more stringent Bonferroni correction (Figure 2A, Supp Fig 2). Three significant differences, including the two most significant comparisons, correspond to the three carcinogens identified in the original study to have specific carcinogen-induced genomic signatures; TCP-liver (*p* = 7*e*^−4^ cosine distance 0.18), VDC-liver (*p* = 3*e*^−5^ cosine distance 0.11) and cobalt-lung (*p* < 1*e*^−5^ cosine distance 0.12), highlighting AMSD’s ability to recapitulate signature-based results. There were also many other carcinogens that had no characteristic signature, but appeared to cause stark shifts in mutation spectra, as evidenced by high cosine distance (e.g. bromochloroacetic acid liver: *p* = 2*e*^−4^ cosine distance 0.22). AMSD also flagged the significance of some more subtle difference in mutation spectra, as evidenced by low cosine distance (e.g. oxazepam liver: *p* = 4*e*^−4^ cosine distance 0.035).

AMSD reveals which exposures significantly perturb the mutation spectrum, but it does not tell us why spectra differ between exposures. To investigate why spectra differ, it is helpful to compare the spectra themselves and then apply signature fitting (Supp Fig 3, Supp Fig 4). As representative examples, we picked one high cosine distance tumor type that was flagged by signature analysis, TCP-exposed liver, and one low cosine distance tumor type that was not flagged by signature analysis, oxazepam-exposed liver (Figure 2B). Visualizing signature exposures for each tumor as stacked bar plots, a common method for displaying signature fitting results, we see TCP tumors have a markedly different signature profile than spontaneous liver tumors, reflecting the signature 12 and 19 exposures that were highlighted in the original study (Figure 2C,D). The corresponding human versions of these two signatures do not have a known etiology, though this result in mice indicates that TCP-like compounds may directly or indirectly affect these mutational processes.

For oxazepam, differences in signature exposure fractions are not obvious (Figure 2C), but signature decomposition appears to be masking mutation spectrum differences that exist between oxazepam-exposed and unexposed tumors (Figure 2B). The signature exposure fraction was not significantly different for any individual signature, either by Wilcoxon rank sum test or two-tailed *t*-test with unequal variance (Figure 2E), leaving the possibility that instead of introducing new mutational processes oxazepam might be shifting tumor cell growth/metabolism or DNA repair in ways that perturb the relative rates of endogenous mutational processes.

Interestingly, chemicals that induce significant mutation spectrum divergence by AMSD are only marginally more likely to be positive on an Ames test, a bacterial assay used to assess the mutagenic potential of chemical compounds (5/12 versus 3/17, *p* = 0. 2, Fisher’s exact test). This is consistent with our hypothesis that carcinogens affecting mutation spectra do not necessarily introduce new mutational processes. However, chemicals with significant effects on mutation spectra according to AMSD were more likely to have “clear evidence” for carcinogenicity (11/12) than those without a detectable spectra difference (8/17) (*p* = 0. 02, Fisher’s exact test) in the results from the National Toxicology Program Bioassay, which uses rodent studies to determine the strength of the evidence that each chemical is carcinogenic. Ultimately, our results indicate that many more carcinogens may affect mutation spectra than previously detected by signature fitting methods, and that AMSD is sensitive to underlying differences in mutation spectra even in experimental data sets with relatively small sample sizes.

One known human carcinogen that has not been linked to a difference in mutational signatures is asbestos, a material used in construction that is known to cause mesothelioma^10–12^. However, Mangiante et al. detected molecular differences that correlate with asbestos exposure, such as the expression of mesenchymal and neoangiogenesis-related genes^12^, which could indirectly influence mutation spectra in subtle ways that signature analysis may not be powered to detect. Though we do not detect a difference in SBS spectra between mesothelioma tumors with (*n* = 75) and without (*n* = 28) known professional asbestos exposure in the Mangiante et al. data set (*p* = 0. 67), we do detect a significant difference in copy number variant (CNV) spectra (*p* = 0. 018) (Supp Fig 5). Similar to SBS, CNV spectra can be generated by classifying CNVs into different types by loss-of-heterozygosity status, total copy number state, and segment length. This asbestos-associated CNV difference is largely driven by high-CNV outliers in the unexposed group with high CN18 (COSMICv3.1^13^ copy number signature of unknown etiology), and is no longer significant (*p* = 0. 21) when all samples’ spectra are weighted equally to diminish the contribution of outlier samples. However, CN9 (diploid chromosomal instability) is higher in professionally exposed patients regardless of how spectra are weighed, so a portion of the signal may result from this contribution as well. This result highlights that AMSD can be applied to any mutation state space, not just SBS in 3mer contexts, and can detect high-mutation outlier biases when samples are weighted by mutation counts.

### Genetic ancestry associations with tumor mutation spectra

One open question in cancer and evolutionary biology is whether endogenous mutational processes have different biases in populations with different genetic ancestry. Differences in inherited germline mutation spectra have been identified in humans^14,15^, and the same genes and exposures that cause these differences might also impact somatic mutagenesis^16^. Though some ancestry effects on tumor mutagenesis have been identified using mutational signature-based methods^17^, to our knowledge there has not been a comprehensive screen for mutation spectrum differences between tumors from different ancestry groups.

The Cancer Genome Atlas (TCGA) is a large compilation of exonic somatic mutation calls for thousands of tumors across 33 cancer types^18^. The data set is majority European ancestry (EUR, 83%), but for many cancer types there are sufficient samples of African (AFR, 9.6%) and East Asian (EAS, 6.6%) ancestry to test for ancestry differences in mutation spectrum composition using AMSD^17^. Although this European bias is not ideal for research equity or for power to detect differences, TCGA is still more diverse than other large cancer sequencing cohorts such as PCAWG^19^. Of note, when interpreting these results from an observational study like TCGA, as opposed to a controlled experimental study like the mouse carcinogen exposures, significant differences from AMSD imply an association, not necessarily genetic causation. In addition to genetic effects caused by allele frequency differences between ancestries, tumor mutation spectra might also be impacted by patient sampling biases or geographic, socioeconomic, or cultural differences in environmental exposures.

**Figure 3.**
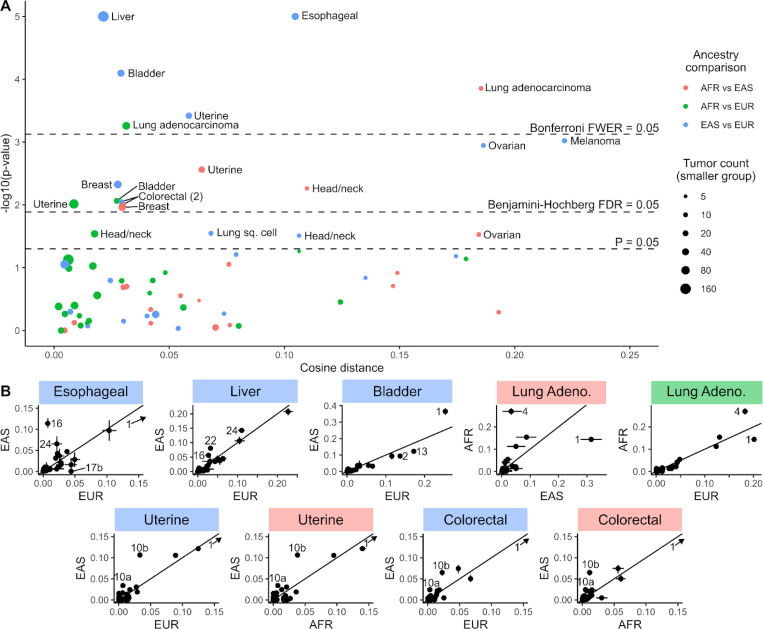
Mutation spectrum differences associated with genetic ancestry. (**A**) Volcano plot of AMSD results comparing TCGA tumors within each cancer type by genetic ancestry. Colors denote pairwise ancestry comparison (AFR vs EAS = red, AFR vs EUR = green, EAS vs EUR = blue), and sizes denote the number of tumors in the smaller of the two groups in each comparison. Dashed lines correspond to significance thresholds: individual *P* = 0.05 threshold, Benjamini-Hochberg corrected false discovery rate (FDR) adjusted threshold, and Bonferroni corrected family-wise error rate (FWER) adjusted threshold. (**B**) Signature exposure fractions in the aggregate spectra for each ancestry group for 9 significantly different comparisons discussed in the text, with each dot denoting a COSMIC v3.2 SBS signature. Diagonal line denotes 1:1 ratio, with the largest deviating signatures labeled. Error bars denote 95% confidence fitting error interval. SBS1 was cropped out from some plots when it was so high it made interpretation of other signatures difficult, and is indicated with an arrow (see Supp Fig 7 for full plots).

We used AMSD to compare spectra for each tumor type for which both ancestries had at least 5 samples. Of 67 comparisons, 16 remained significantly different after Benjamini-Hochberg correction and 6 remained significantly different after Bonferroni correction (Figure 3A, Supp Fig 6). To interpret what factors may be driving significant differences between ancestries, again we turn to signature analysis. However, we note that the resolution to fit signatures in individual samples is limited by the low mutation counts of whole exome sequencing data (median of 168 mutations/tumor across TCGA). Due to this limitation, we fit signatures to the aggregate spectrum for each sample grouping and compare for the largest differences in signature exposure (Figure 3B, Supp Fig 7). SBS1, attributed to endogenous deamination of 5-methylcytosine, was the highest signature exposure in most cancer types and was often over-represented in one ancestry. We attribute this trend to SBS1’s role as a steady background mutational process, which will be lower as a fraction of total mutations in the presence of added non-endogenous exposures since mutational fractions will always add up to 1 (though the same trend does not hold for the other background process, SBS5). Thus, we focus on other signatures to interpret the processes driving ancestry-associated differences.

Lung adenocarcinoma was the only cancer type where we observed significant differences in mutation spectra across multiple population comparisons after Bonferroni correction (AFR vs EAS: *p* = 1*e*^−4^, AFR vs EUR: *p* = 6*e*^−4^). The main driver of this difference appears to be higher AFR exposure to COSMIC SBS4, a signature closely associated with smoking that mainly consists of C>A mutations (Figure 3B, Supp Fig 8). The difference in SBS4 exposure between AFR and EUR ancestry has been noted before, though notably this was not attributed to smoking rates, as AFR patients actually appear to smoke significantly less than EUR patients^20^. Among lung adenocarcinoma patients from this study specifically, AFR patients smoked an average of 19.6 pack-years versus 29.8 pack-years in EUR patients (*p* = 0. 004, two-sample, two-sided unequal variance t-test). This counterintuitive result suggests that this difference may in fact be due to a difference in SBS4 susceptibility, perhaps due to differences in the ability to repair smoke-induced DNA adducts or the immune/inflammatory response to smoking. Some studies have reported individuals of African ancestry to be more susceptible than individuals of European ancestry to the carcinogenic effects of tobacco smoke^21^, which might lead to an association between SBS4 exposure and African ancestry among lung cancer patients. This finding highlights AMSD’s ability to detect a known mutation difference between AFR and EUR lung cancers and extends its generality by also detecting a difference between AFR and EAS lung cancers, despite low EAS sample size (*n* = 9).

Uterine cancer was another tumor type where AMSD detected a difference across multiple ancestry comparisons (EAS vs EUR: *p* = 4*e*^−4^, AFR vs EAS: *p* = 3*e*^−3^, AFR vs EUR: *p* = 1*e*^−2^, both significant at a Benjamini-Hochberg FDR of 0.05). The main driver for the difference between EAS and EUR/AFR appears to be higher EAS exposure to SBS10a/b, signatures associated with polymerase epsilon exonuclease domain mutations consisting mainly of TCT>TAT and TCC>TTC mutations (Figure 3B, Supp Fig 9). Interestingly, we observe the same signal in colorectal cancer (EAS vs EUR: *p* = 9*e*^−3^, AFR vs EAS: *p* = 1*e*^−2^, both significant at a Benjamini-Hochberg FDR of 0.05), the other cancer type in which SBS10a/b are regularly detected (Supp Fig 9). The increase in SBS10 exposure can be attributed to a higher proportion of tumors with this exposure in EAS for both uterine and colorectal cancer (proportions of uterine cancers with SBS10a/b are 9/109 AFR (8%), 7/29 EAS (24%), 20/375 EUR (5%), while proportions of colorectal cancers with SBS10a/b are 0/57 AFR (0%), 2/12 EAS (17%), 6/339 EUR (2%)). Previous studies have noted a higher prevalence of POLE mutations in patients of East Asian ancestry^22–24^, but to our knowledge this pattern has not been detected using the prevalence of SBS10a/b.

The two most significant mutation spectrum differences are esophageal cancer and liver cancer, both when comparing EAS to EUR (*p* < 1*e*^−5^, Supp Fig 8). Notably, both of these cancer types have been reported to occur at disproportionately high rates in Asian countries^25^. EAS had higher SBS16 exposure in both cancer types, a signature with unknown etiology, though some studies have linked SBS16 to alcohol consumption^26–28^. Rates of both esophageal cancer incidence and SBS16 exposure both have been found to vary significantly with geography^29,30^. These SBS16 differences may be linked to aldehyde dehydrogenase 2, an enzyme involved in alcohol metabolism with a nonfunctional allele common in EAS but rare in other ancestries (rs671)^27,31–33^. This might exhibit a similar effect as SBS4 in AFR, increasing mutational exposure despite lower alcohol/tobacco use^34^. EAS also has higher SBS24 (associated with aflatoxin, a mold that grows on crops) in both cancer types, and higher SBS22 (associated with aristolochic acid, a plant compound found in herbal medicines and contaminated wheat) in liver cancers, potentially pointing toward differences in diet. EUR esophageal cancers have higher SBS17b, which has been linked to fluorouracil chemotherapy treatment and is virtually absent from EAS esophageal cancers. This may be due in part to dihydropyrimidine dehydrogenase deficiency, which increases the risk of fluorouracil toxicity and is more common in EUR than EAS^35^. However, only a small proportion of esophageal cancer patients were treated with fluorouracil (8/126 EUR, 3/44 EAS), so this effect is likely related to other putative causes of SBS17b, such as reactive oxygen species.

In addition to these cancer types, we observed significant differences in the context of bladder cancer (EAS vs EUR and AFR vs EUR: SBS2/13 - APOBEC cytidine deaminase activity), melanoma (EAS vs EUR: SBS7a/b - ultraviolet light exposure), ovarian cancer (EAS vs EUR: SBS49 - possible sequencing artifact), head and neck cancer (AFR vs EAS: SBS87 - Thiopurine chemotherapy treatment), and breast cancer (EAS vs AFR and EAS vs EUR: SBS2/13 - APOBEC), summarized in Supp Fig 7. Overall, these findings indicate that ancestry-associated mutation biases exist but are highly tumor-type dependent. These results also highlight the power of AMSD as a screening tool, with signature analysis interpreting the mutational processes behind significant results.

## Discussion

In this study, we apply the statistical framework of the AMSD permutation test to tumor datasets from mice and humans, revealing that carcinogens and genetic ancestry can affect cancer mutation spectra in situations that previously went undetected using standard signature-based methods. Although conclusively explaining the drivers behind these trends is beyond the scope of this paper, we are able to propose likely drivers through targeted mutational signature analysis when AMSD detects a significant difference. These examples demonstrate the power of using AMSD as a hypothesis-testing or screening tool alongside established mutational signature approaches: AMSD directly tests whether mutation spectra differ between groups without requiring prior assumptions about the decomposition of spectra into signatures, while mutational signature analysis reveals complementary information about which biological processes likely contribute to the observed mutation spectrum differences. Used together, these methods offer a more complete picture of how mutational processes vary across biological conditions, enabling both unbiased detection and mechanistic interpretation of differences in genomic integrity.

Although many carcinogens induce very distinctive mutational signatures^9^, the majority of carcinogens appear to cause more subtle shifts in mutation spectrum composition, as previously highlighted by Morrison et al. using a metaanalysis of mutational signature dosages^36^. For many carcinogens, AMSD detects subtle differences associated with exposure even when the etiology of these differences remains unresolved. Rather than being attributable to the introduction of new mutational processes, we suspect these differences are caused by perturbing endogenous processes via inflammation, immune response, microenvironment, or metabolism/growth rates^37^. Notably, these carcinogens would not necessarily cause a detectable increase in tumor mutation burden if they made small but consistent shifts in endogenous mutational processes, perhaps substituting a higher chemical-associated mutation load for a lower load of age-associated mutations if chemical exposure causes cancer to develop at a younger age. Morrison et al. found carcinogen exposures increase the diversity of mutational signatures within tumors and the homogeneity of signature activity across tumors^36^, which may contribute to the mutation spectrum shifts we observe here. These findings underscore that carcinogen-induced changes to mutation spectra can arise through complex and indirect biological pathways, highlighting the value of sensitive, assumption-free methods like AMSD for detecting these patterns.

Despite TCGA’s European bias and the limitations of exome-sequencing data, we were able to detect significant mutation spectrum differences between continental ancestry groups across multiple cancer types. While prior work has revealed differences in cancer incidence and mutational burden across populations^17^, our results suggest that the underlying mutation processes themselves may also differ in subtle but systematic ways. These differences may be directly mediated by genetic causes of cancer risk, or they might reflect environmental differences that covary with genetic ancestry^38^. Demographic differences between who suffers from cancer in different populations might also play a role, for example if wealthier populations experience later age of cancer onset. As larger, more diverse cancer datasets become available, methods like AMSD will be critical for rigorously testing whether mutational processes vary across populations and identifying underlying factors contributing to disparities in cancer risk and outcomes.

Though our main analyses apply AMSD to the most commonly used mutation spectrum state space and data type – SBS 3mer spectra ascertained using bulk tumor sequencing – mutation spectra can be generated and compared with AMSD in other contexts. This approach is straightforwardly applicable to spectra data from double base substitutions, insertion/deletions, structural variants, and copy number alterations (as demonstrated in mesothelioma), as well as extended SBS sequence contexts, such as 1mer, 5mer, transcribed/untranscribed strand and genic/intergenic contexts. Other sequencing methods, such as single-cell, multi-region, or spatial transcriptomics, provide greater intra-tumor resolution in which AMSD could test for cases when spectra vary across time and space in individual tumors^39^. AMSD can also be applied in a GWAS-like scan for mutator alleles, similar to its initial application in recombinant inbred mice^8^, although we caution that preliminary attempts to calculate AMSD-based mutation spectrum associations with millions of variants across the human genome faced a heavy multiple testing burden, so we would recommend using it on target loci or on datasets with more resolution than TCGA. Finally, AMSD is not limited to cancer datasets and could be applied in other capacities, such as germline mutation rate evolution^14^ or somatic mutations in healthy tissues^40^ when sufficient numbers of mutations or samples are available.

In conclusion, AMSD is a versatile and powerful framework for detecting differences in mutation spectra across diverse biological settings. As sequencing technologies advance and datasets grow larger, tools like AMSD will be essential for identifying subtle shifts in mutational processes that may be overlooked by mutational signature analysis alone. Whether applied to population-level studies, environmental exposure screens or testing individual hypotheses, AMSD provides a statistically grounded method with minimal underlying assumptions that can test for mutation spectrum associations to advance our understanding of mutagenesis and cancer evolution.

## Methods

### Data and code availability

The Aggregate Mutation Spectrum Distance permutation test is implemented as the R package “mutspecdist”, available at https://github.com/sfhart33/mutspecdist. All analyses in this study use publicly available datasets, and figures and results can be reproduced using the code available at https://github.com/sfhart33/AMSD_cancer_mutation_spectra. Preprocessed mutation spectra are included in the repository, while raw data can be accessed from:

- Mouse carcinogen exposure: https://github.com/team113sanger/mouse-mutatation-signatures/blob/master/starting_data/snvs.rds
- Asbestos exposure: https://github.com/IARCbioinfo/MESOMICS_data/tree/main/phenotypic_map/MESOMICS
- TCGA ancestry metadata: https://gdc.cancer.gov/about-data/publications/CCG-AIM-2020
- TCGA somatic mutations https://gdc.cancer.gov/about-data/publications/mc3-2017

The original implementation of the AMSD as a method for identifying mutator alleles is also available on github: https://github.com/quinlan-lab/proj-mutator-mapping.

### The aggregate mutation spectra distance (AMSD) permutation testing pipeline

As input, the AMSD permutation test takes two matrices of mutation spectra, such as a group exposed to a carcinogen and a control group unexposed to a carcinogen. AMSD then aggregates the mutation spectra within each group into a single spectrum, either by taking the average frequency of each mutation type (the default, which weights all samples equally regardless of mutations per sample) or a sum of the count of each mutation type (resulting in the upweighting of samples with higher mutation loads). AMSD then calculates a distance metric (default = cosine distance) between the two groups. To calculate the significance of this distance, AMSD then randomly reshuffles the samples to create the two control groups (same number of samples in each group as the original grouping), computes the aggregate mutation spectra and distance associated with this new grouping, and repeats this reshuffling process for a specified number of permutations (default = 1000) to create a null distance distribution. Then AMSD compares the observed distance to the null distribution, outputting a *p*-value corresponding to the fraction of random reshufflings that produce a distance greater than or equal to the observed distance. This observed versus null comparison can be visualized as a histogram or violin plot, as seen in the paper figures.

### AMSD power analysis with simulated data

In order to test AMSD’s ability to detect spectrum differences, we built in a framework to simulate spectra sampled from pre-set mutational signature exposures. The simulation framework randomly samples mutations at probabilities corresponding to their frequencies in supplied signatures (COSMIC v3.2). For the test run in Supp Fig 1, we set the control group baseline to 30% SBS1, 60% SBS5, and 10% SBS18 to simulate endogenous mutational processes. We then simulate an “exposure” group, in which we sampled from the same baseline distribution of mutational signatures and then sampled additional mutations from one additional signature. We varied four parameters (listed below), running 100 simulations for every combination of these parameters. We then compared the “control” and “exposure” spectra groupings for each simulation using AMSD and for each parameter combination reported the fraction of the 100 simulations for which AMSD detected the difference between the control and exposure group at a significance level of *p* < 0.05.

- Baseline mutations per sample:
  - 50 to simulate a whole exome sequencing data set
  - 2500 to simulate whole genome sequencing
- “Exposure” signature added
  - SBS2 to simulate a common “spikey” signature (small number of high-frequency trinucleotides)
  - SBS40 to simulate a common “flat” signatures (relatively equal frequencies of mutation probabilities across many trinucleotide contexts)
- Extra mutations from “exposure” signature, as a % of control mutation count
  - 2% to simulate a weak exposure (e.g. 50 extra mutations for the 2500 baseline mutation group)
  - 5%
  - 10%
  - 20% to simulate a strong exposure
- Number of samples per group
  - 5 to simulate a small experimental study (e.g. Riva et al.)
  - 25
  - 125
  - 625 to simulate large observational study (e.g. breast cancers in TCGA)

### Mouse carcinogen analysis

We downloaded SNV mutation calls from Riva et al.^7^ and tallied trinucleotide context mutation counts using Helmsman^41^ (v1.5.2) against *M. musculus* (house mouse) genome assembly GRCm38 (mm10). We then used AMSD to compare each carcinogen-exposed tumor type to the control set of spontaneously arising tumors from the same tissue type (liver or lung tumors). We used the default AMSD settings, weighting all sample spectra equally, but with 100,000 random resamplings for the null expectation. Results comparing observed cosine distance to the null distribution are available as violin plots for each AMSD comparison (*n* = 29) in Supp Fig 2. To correct for multiple testing, we used Bonferroni and Benjamini-Hochberg adjusted *p*-values. To show all results on same plot (Figure 2A), we show a Bonferroni Correction threshold ( − log_10_ (0. 05/*n*)), and an estimated Benjamini-Hochberg correction threshold from linear regression of − log_10_ (adjusted *p*-values) against − log_10_ (unadjusted *p* -values) (*R*^2^ = 0 . 994 β = 1 . 32) for a threshold of −log_10_ (0. 05 ) × β.

In order to interpret potential mutational signature drivers behind significant results, we also downloaded the mutational signature exposures from Riva et al.. In brief, they extracted mutational signatures de novo, compared them to COSMIC signatures to identify similar human signatures, and fit each sample to the mouse mutational signatures, keeping only signature exposures that were at least 5% signature exposure fraction. We compared signature exposures (Figure 2D,E) using both the Wilcoxon rank sum test and the two-tailed *t*-test with unequal variance. For all signature comparisons involving the highlighted examples of oxazepam and TCP, both tests were in agreement for all signatures (both either *p* < 0. 05 or *p* > 0. 05).

### Asbestos exposure analysis

We downloaded SNV, CNV, and SV mutation calls from Mangiante et al.^12^ and tallied these respective mutation counts in standard COSMIC format using Helmsman^41^ (v1.5.2) for SNVs or SigProfilerMatrixGeneratorR^42^ (v1.2) for CNVs and SVs. For each mutation type, we grouped tumors by whether the patients had been professionally exposed to asbestos or not professionally exposed to asbestos and ran AMSD with 10,000 random resamplings for the null expectation. We ran both AMSD methods for each - weighting all sample spectra equally with aggregate mutation spectra means and weighting samples by mutation count with aggregate spectra sums. We also downloaded copy number mutational signature exposures from Mangiante et al., calculating the mean of each signature exposure fraction (for “means” - weighted evenly), count (for “sums” weighted by mutation counts), or count after dropping outlier samples with >500 copy number variants (“sums, no outliers”) for the exposed/unexposed groupings. We also calculated the standard error of the mean $\left(\text{σ}/\sqrt{n}\right)$ for each mean to estimate uncertainty, plotted in Supp Fig 5G–I.

### TCGA ancestry analysis

We downloaded TCGA SNV mutation calls from Ellrott et al.^18^ and tallied trinucleotide context mutation counts using Helmsman^41^ (v1.5.2) against the human genome assembly GRCh37 (hg19). We then grouped tumors by consensus superpopulation ancestry calls from Carrot-Zhang et al.^17^, removing patients that were not majority African (AFR), East Asian (EAS) or European (EUR) ancestry due to low South Asian and Admixed American sample sizes. We also removed tumors with <10 mutations so that these samples would not be evenly weighted with tumors with more mutation type resolution. We then ran pairwise comparisons using AMSD for all cancer types for which both ancestries in the comparison had at least 5 samples. For the full list of comparisons (*n* = 67), see results in Supp Fig 6, with tumor types following standard TCGA abbreviations. We used the default AMSD settings, weighting all sample spectra equally, but with 100,000 random resamplings for the null expectation. To correct for multiple testing, we used Bonferroni and Benjamini-Hochberg adjusted p-values. To show all results on same plot (Figure 3A), we show a Bonferroni correction threshold ( − log_10_ (0. 05/*n*)), and an estimated Benjamini-Hochberg correction threshold from linear regression of − log_10_(adjusted p-values) against − log_10_(unadjusted p-values) (*R*^2^ = 0 . 999, β = 1 . 45) for a threshold of ( − log_10_ (0. 05) × β.

In order to identify which mutational signatures were likely to be driving significant AMSD differences, we fit aggregate spectra, grouped by ancestry and cancer type, to the COSMIC SBS v3.2 mutational signature database. Since mutation calls for each sample were limited to exons, many samples had a low number of mutations per sample and thus would not be suitable for calling mutational signatures on a per sample basis. Thus, we aggregated spectra using the same method as used for AMSD, weighing all samples evenly to get an average spectrum, converted from mutation fraction to mutation counts by multiplying by the total number of mutations, and then fit signatures using sigfit^43^ (v2.2.0) using the “opportunities = human-exome” option to correct for the exon-only mutations in this set. We chose sigfit for signature fitting because; 1) sigfit outputs 95% confidence fitting error for each signature to capture uncertainty for each signature exposure, 2) sigfit does not have prior assumptions of which supplied signatures may be present/absent in the spectrum, 3) sigfit returns all signatures as non-zero fractions, even when the fractions are very small or have high error, which is appropriate when fitting the aggregate spectrum from many tumors, some of which may have rare signatures. Finally, we compared the fitting outputs for each comparison, filtering for signatures with the largest exposure difference between ancestries, as shown and labeled in Figure 3B and Supp Fig 7.

## Supplementary Material

Supplement 1

## Figures and Tables

**Table 1.** Cases where mutational signature differences may lack statistical or biological significance

Situation	Description	Example
Low mutation counts	Low resolution to fit signatures when mutations count < mutation types	Whole exome seq.
Similar signatures	Fitting can confuse similar signatures. Complicated by noise, other signatures	SBS17b vs 28
“Flat” signatures	More difficult to confidently resolve “flat” than “spiky” signatures	SBS3, 5, 40
Missing signatures	An unknown signature may be forced to fit known signatures	
Signature variability	Signatures will vary based on methodology and input data set	COSMIC v2 vs v3
Signature overlap	Splitting of previous signatures reveals some are multiple processes	SBS7➔7a/7b/7c/7d
Overfitting	False positive signatures from noise and fitting too many signatures	100+ COSMIC sigs
