# Supplementary material for: A signature-agnostic test for differences between tumor mutation spectra reveals carcinogen and ancestry effects
Source: bioRxiv. 2025 May 19:2025.05.15.654154. Preprint. [Version 1] doi: 10.1101/2025.05.15.654154 (PMC12139916; doi:10.1101/2025.05.15.654154)
Supplement: Supplement 1 [file NIHPP2025.05.15.654154v1-supplement-1.pdf]

bioRxiv preprint doi: https://doi.org/10.1101/2025.05.15.654154; this version posted May 19, 2025. The copyright holder for this preprint (which was not certified by peer review) is the author/funder, who has granted bioRxiv a license to display the preprint in perpetuity. It is made available under aCC-BY 4.0 International license.

Supplementary Figures

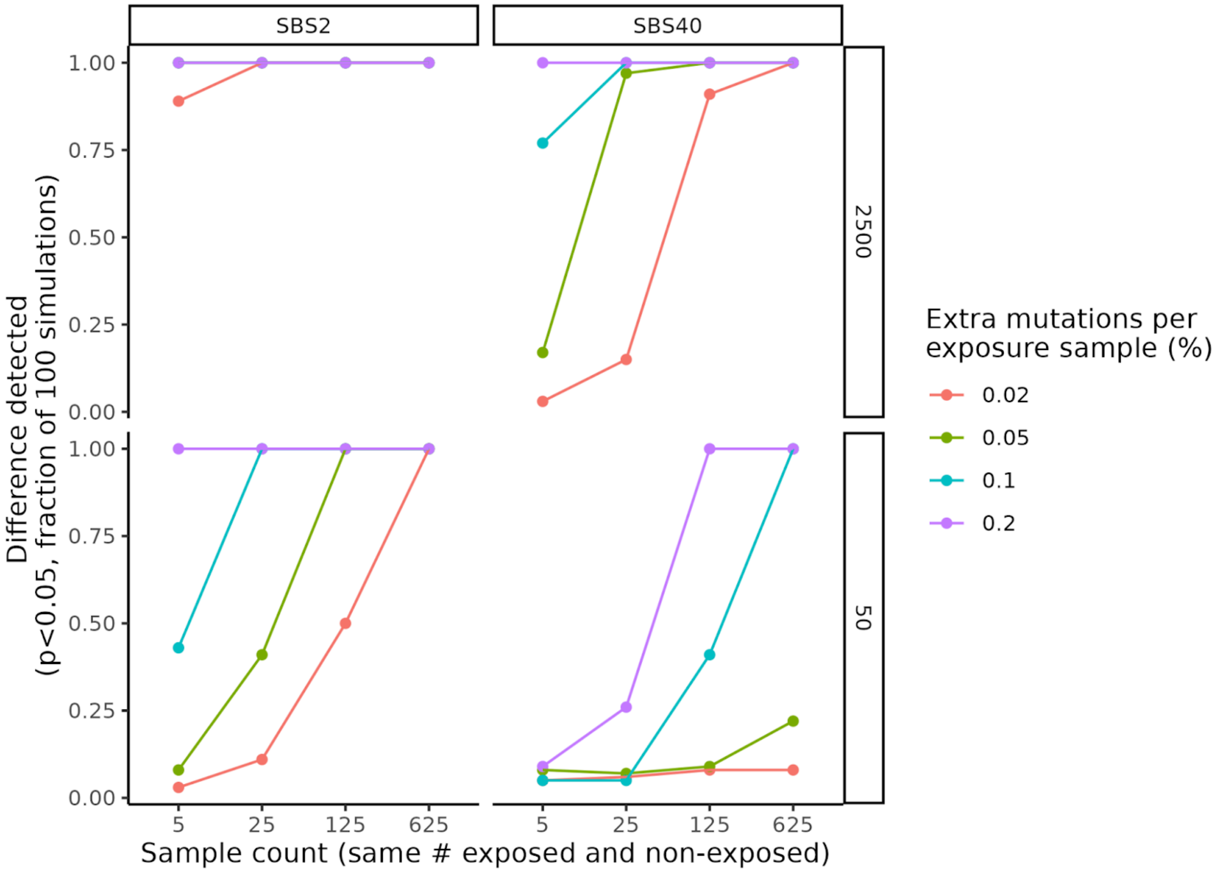

Supp Fig 1. AMSD's power of detection in simulated data sets
The fraction of trials out of 100 in which a significant difference between two groups was detected given four variable parameters: variable signature present in one group ("spiky" SBS2 or "flat" SBS40: left and right columns), number of mutations per sample (2500 to represent whole genome sequencing, 50 to represent whole exome sequencing: top and bottom rows), number of samples per group (5- 625 to represent the range from a small experimental study like Riva et al. to a large observational study like TCGA: x-axes), and number of extra mutations per sample in the exposure group as a percentage of total mutations (2-20%: colors).

bioRxiv preprint doi: https://doi.org/10.1101/2025.05.15.654154; this version posted May 19, 2025. The copyright holder for this preprint (which was not certified by peer review) is the author/funder, who has granted bioRxiv a license to display the preprint in perpetuity. It is made available under aCC-BY 4.0 International license.

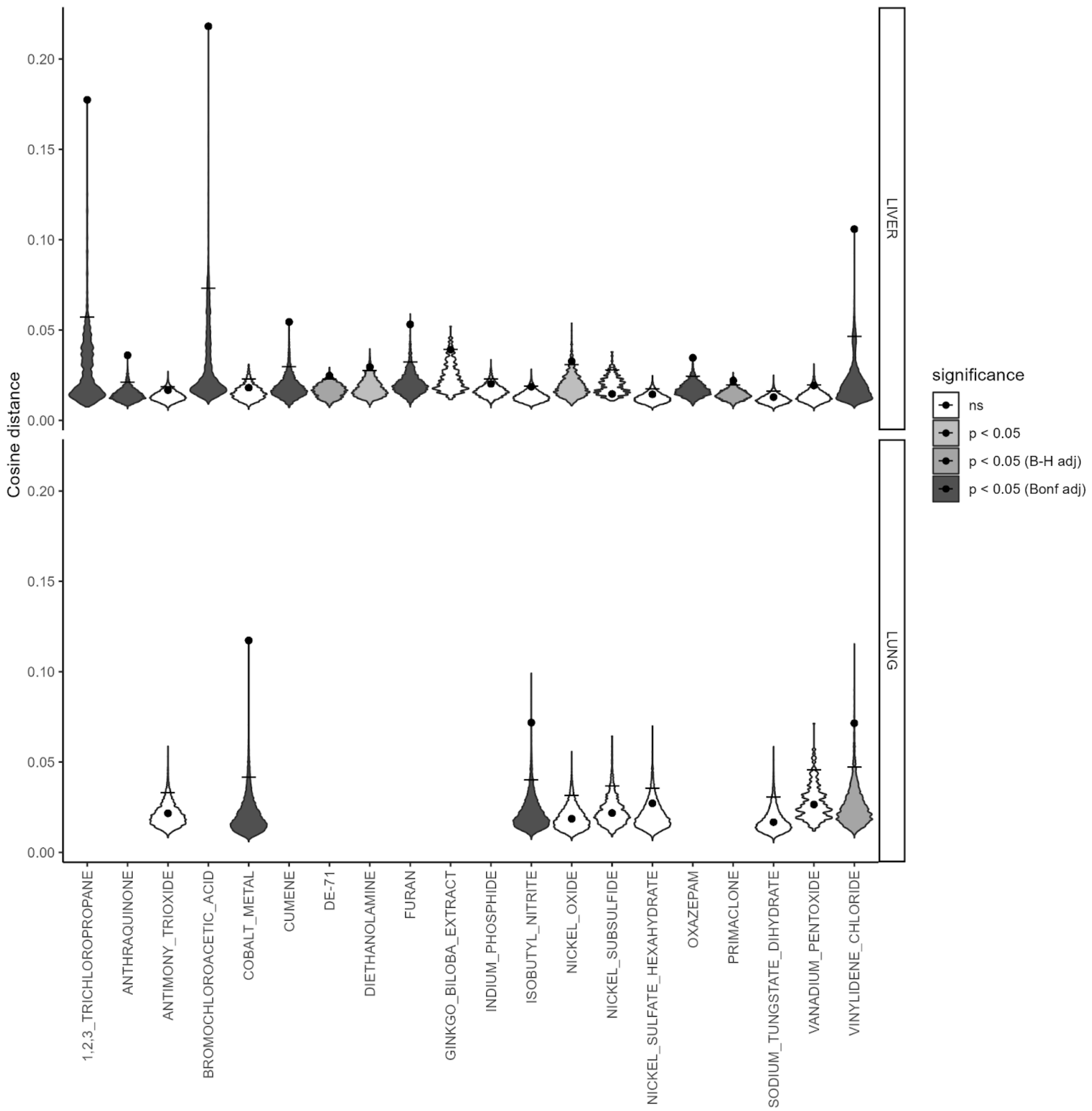

Supp Fig 2. AMSD results for all mouse carcinogen exposures

Violin plots for each AMSD comparison presented in Figure 2A. Violins display the null distribution of random samplings (100,000 each), points display the observed cosine distance between the carcinogen-exposed and spontaneous tumors, and lines represent the 95% quantile p=0.05 threshold. Violin plots are also colored by the corresponding significance threshold (unadjusted, Benjamini-Hichberg adjusted, or Bonferroni adjusted).

bioRxiv preprint doi: https://doi.org/10.1101/2025.05.15.654154; this version posted May 19, 2025. The copyright holder for this preprint (which was not certified by peer review) is the author/funder, who has granted bioRxiv a license to display the preprint in perpetuity. It is made available under aCC-BY 4.0 International license.

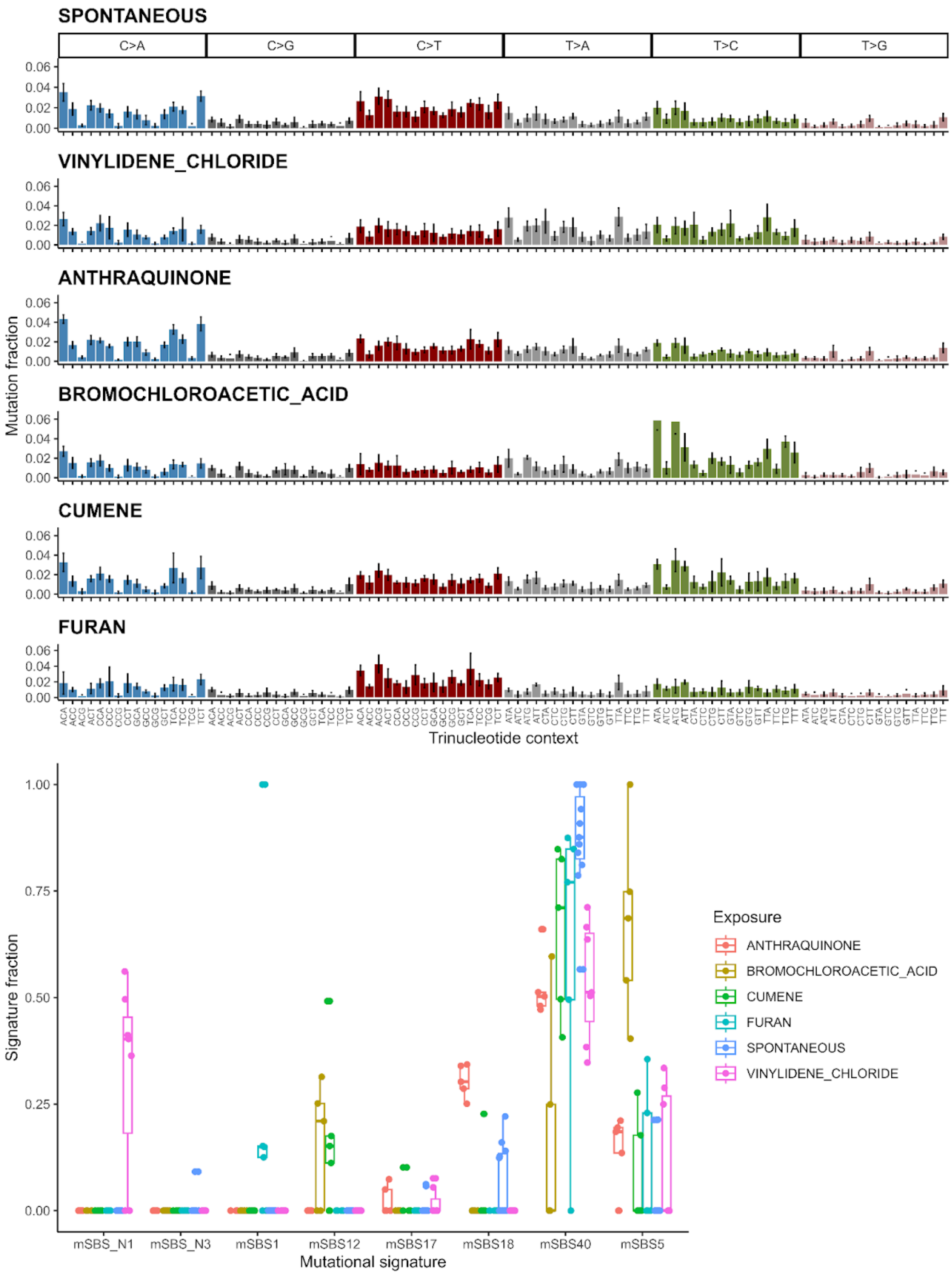

Supp Fig 3. Spectrum and signature results for other significant mouse liver carcinogens
Aggregate mean mutation spectra for mouse liver tumors, with standard deviation in error bars, for spontaneous tumors and carcinogen-exposed liver tumors that were significantly different after Bonferroni correction and not already included in Figure 2 (top). Signature exposures for each sample grouped by exposure and summarized with a boxplot (bottom).

bioRxiv preprint doi: https://doi.org/10.1101/2025.05.15.654154; this version posted May 19, 2025. The copyright holder for this preprint (which was not certified by peer review) is the author/funder, who has granted bioRxiv a license to display the preprint in perpetuity. It is made available under aCC-BY 4.0 International license.

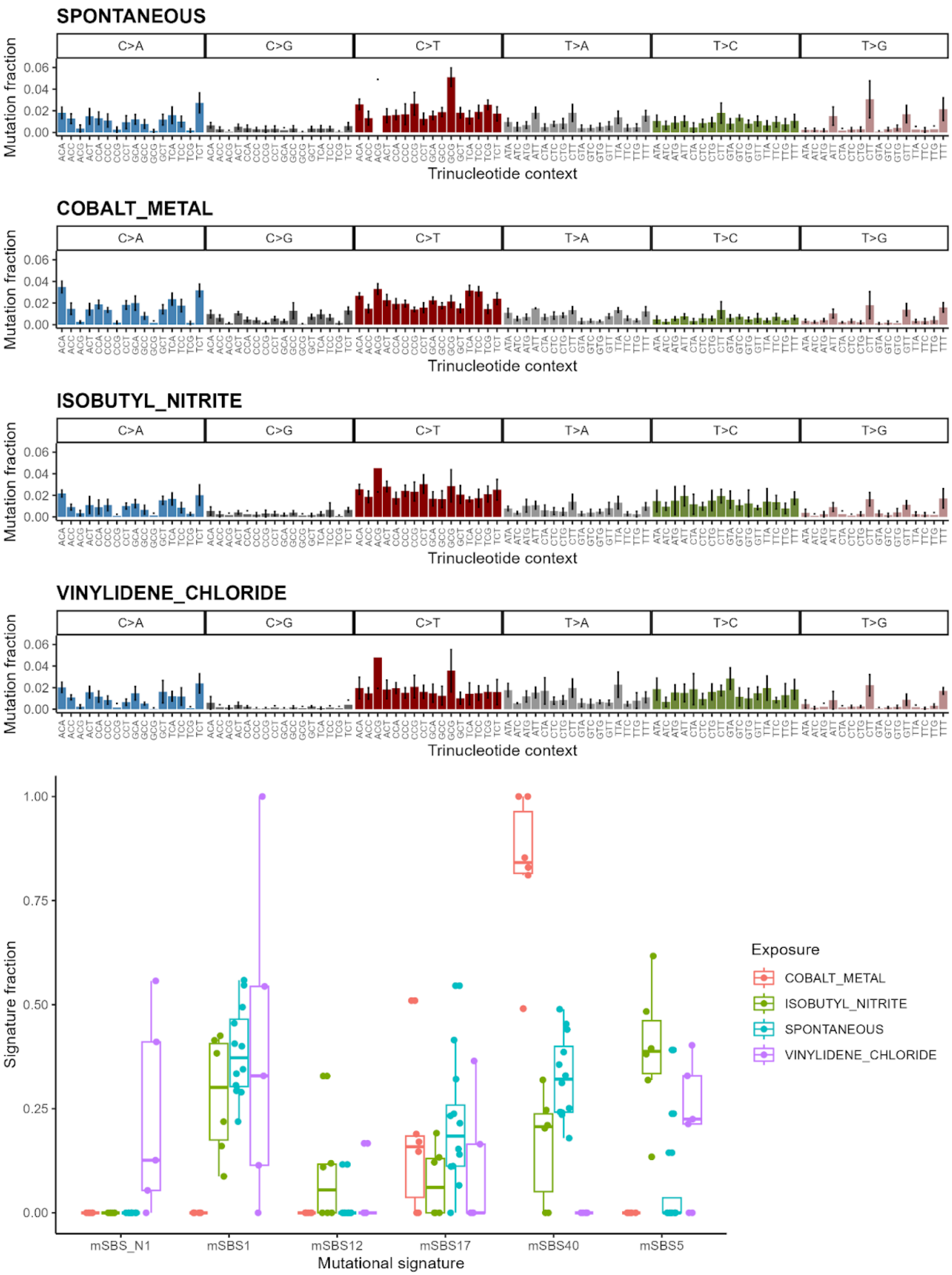

Supp Fig 4. Spectra and signature results for significant mouse lung carcinogens
Aggregate mean mutation spectra for mouse lung tumors, with standard deviation in error bars, for spontaneous tumors and carcinogen-exposed lung tumors that were significantly different after Benjamini-Hochberg correction (top). Signature exposures for each sample grouped by exposure and summarized with a boxplot (bottom).

bioRxiv preprint doi: https://doi.org/10.1101/2025.05.15.654154; this version posted May 19, 2025. The copyright holder for this preprint (which was not certified by peer review) is the author/funder, who has granted bioRxiv a license to display the preprint in perpetuity. It is made available under aCC-BY 4.0 International license.

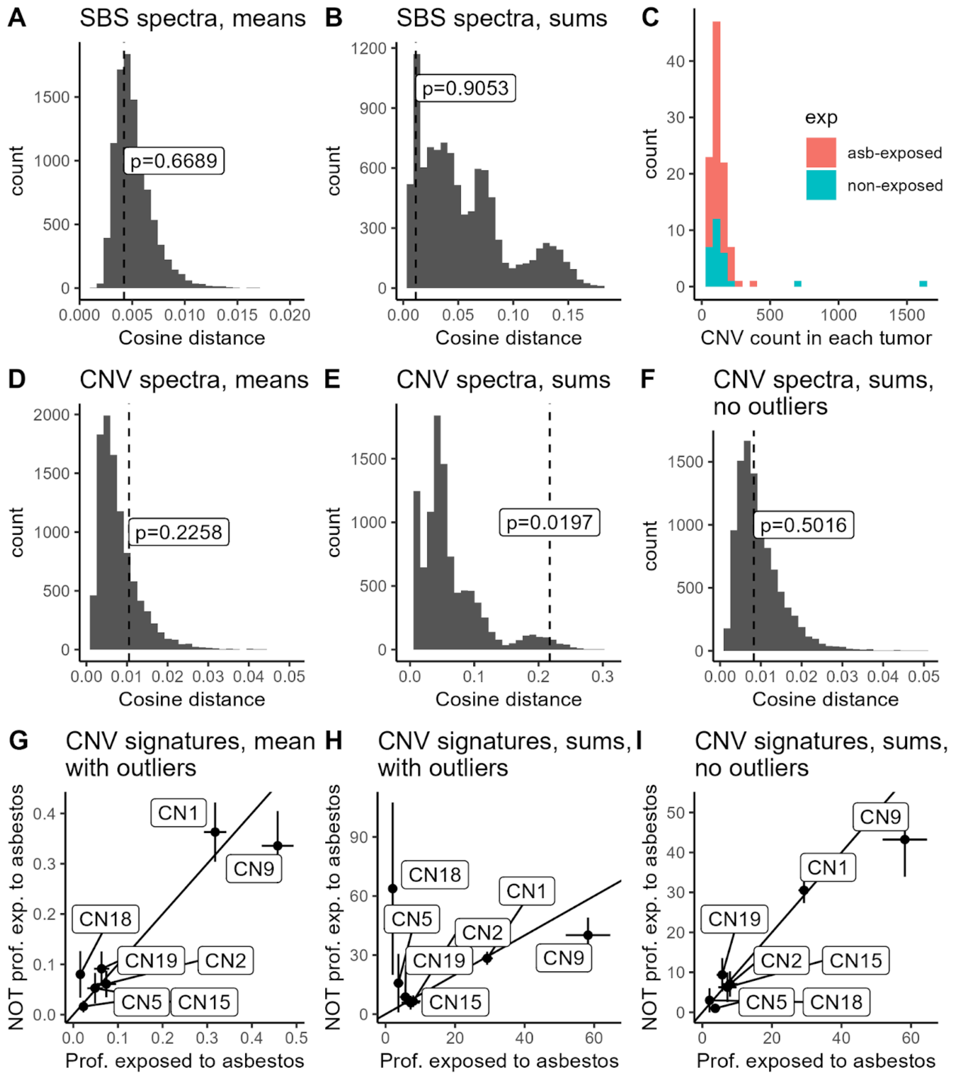

Supp Fig 5. Asbestos exposure influence on CNV spectra

AMSD results for SBS spectra (**A, B**) and CNV spectra (**D, E, F**), comparing mesothelioma spectra for patients professionally exposed to asbestos to those not professionally exposed to asbestos, either weighting all samples equally ("means"), or aggregating all mutations so that samples are weighted by mutation count ("sums"). Histograms show the null distribution expectations from 10,000 random samplings in relation to the real cosine distance between the aggregate spectra and corresponding *p*-value, marked with a dashed line. (**C**) Total CNV count per tumor, showing two high-CNV outliers that were not professionally exposed to asbestos. (**F**) Histogram of AMSD result for CNV sums after dropping >500 CNV outliers. Signature exposures (**G)** or counts (**H, I**) for each COSMIC v3.1 CN signature, comparing the average and standard error of the mean (error bars) by whether or not patients were professionally exposed to asbestos.

bioRxiv preprint doi: https://doi.org/10.1101/2025.05.15.654154; this version posted May 19, 2025. The copyright holder for this preprint (which was not certified by peer review) is the author/funder, who has granted bioRxiv a license to display the preprint in perpetuity. It is made available under aCC-BY 4.0 International license.

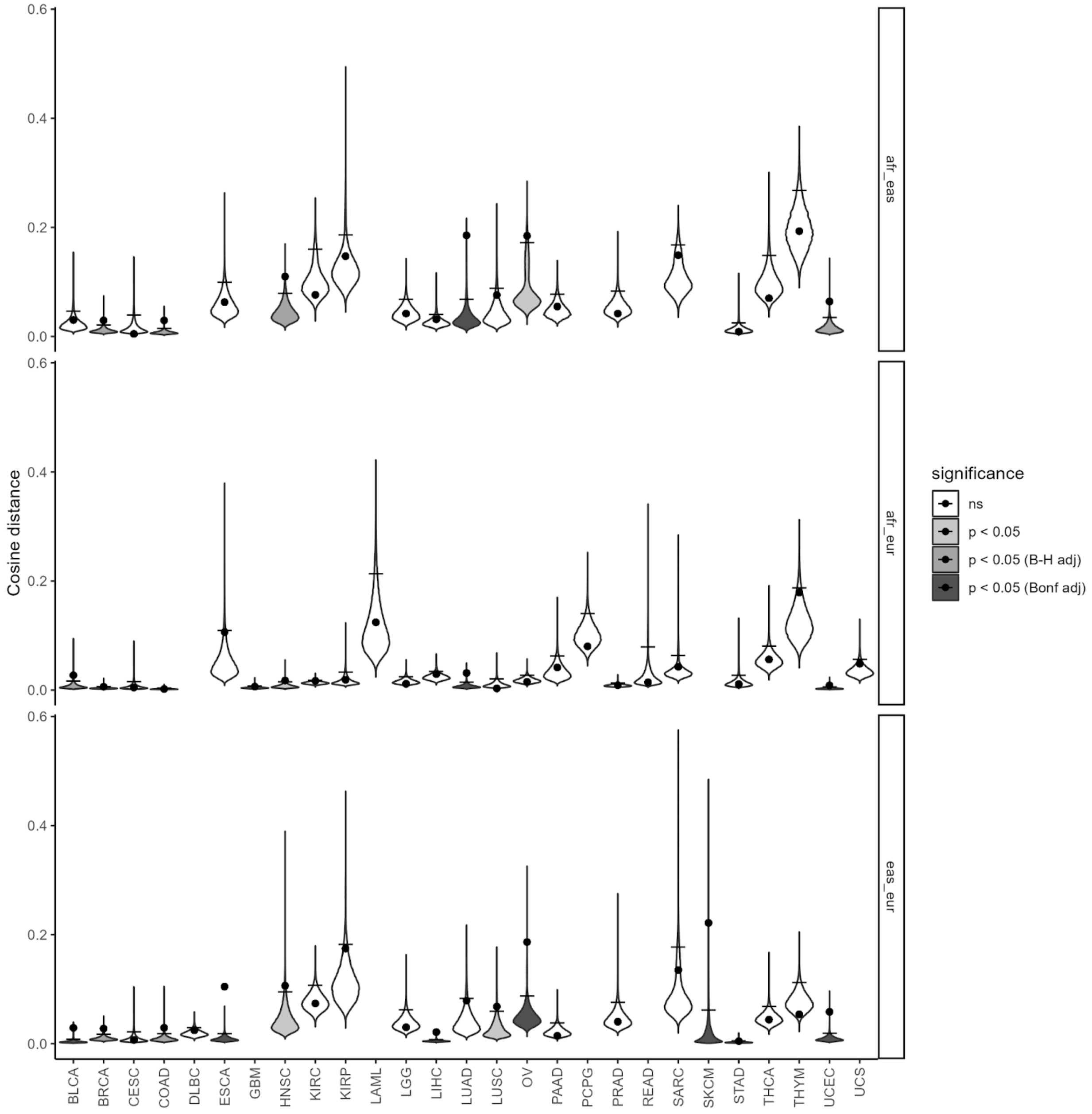

Supp Fig 6. AMSD results for all TCGA ancestry comparisons

Violin plots for each AMSD comparison presented in Figure 3A. Violins display the null distribution of random samplings (100,000 each), points display the observed cosine distance between the carcinogen-exposed and spontaneous tumors, and lines represent the 95% quantile p=0.05 threshold. Violin plots are also colored by the corresponding significance threshold (unadjusted, Benjamini-Hochberg adjusted, or Bonferroni adjusted).

bioRxiv preprint doi: https://doi.org/10.1101/2025.05.15.654154; this version posted May 19, 2025. The copyright holder for this preprint (which was not certified by peer review) is the author/funder, who has granted bioRxiv a license to display the preprint in perpetuity. It is made available under aCC-BY 4.0 International license.

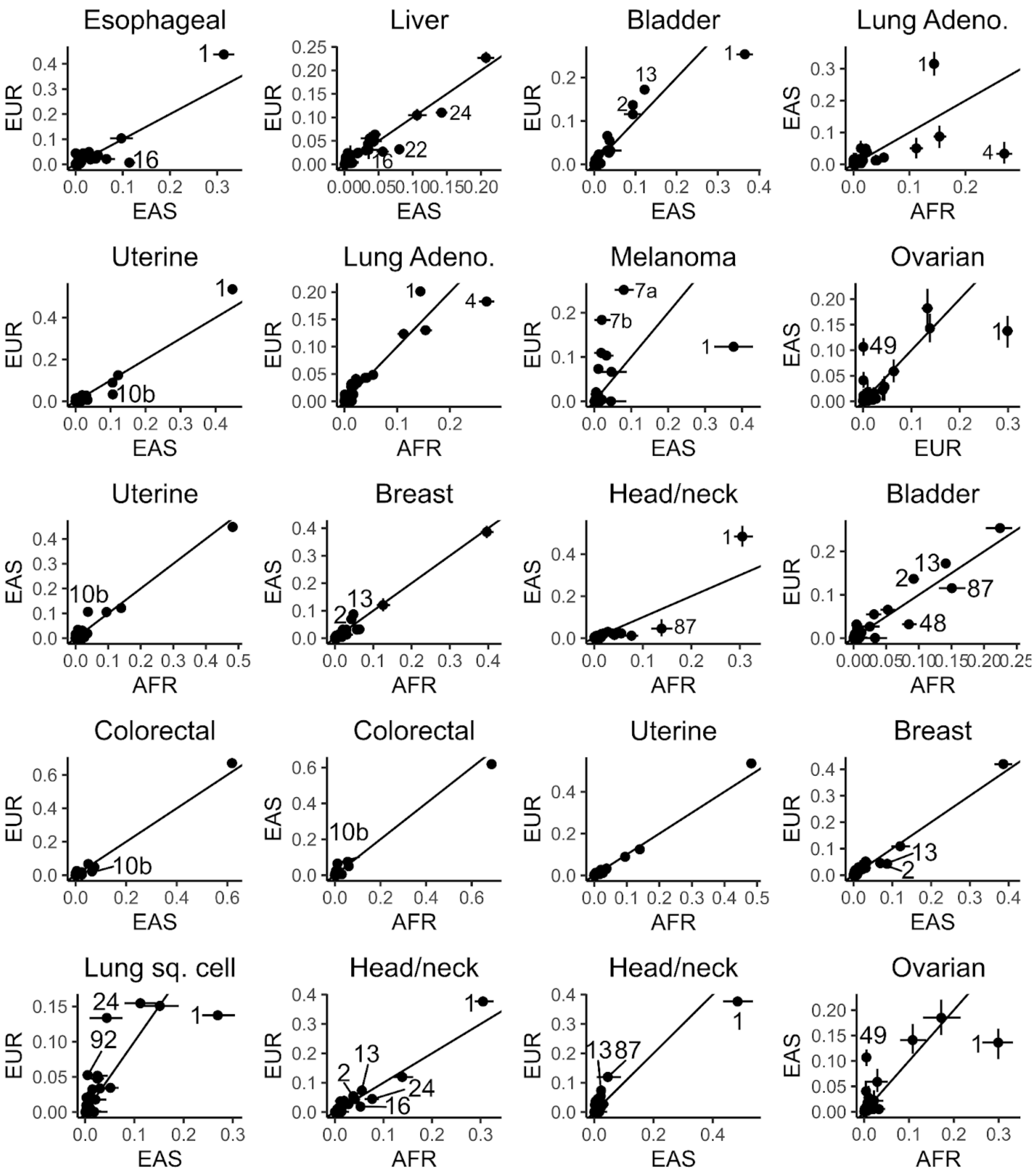

Supp fig 7. Relative signature exposures in tumor pairs with significantly different mutation spectra (*p* < 0.05)
Signature exposures in the aggregate spectra for each ancestry group for tumor types where ancestry-associated mutation spectra differ significantly ($p < 0.05$), with each dot denoting a COSMIC v3.2 SBS signature. Diagonal line denotes 1:1 ratio, with the largest deviating signatures labeled. Error bars denote 95% confidence fitting error intervals.

bioRxiv preprint doi: https://doi.org/10.1101/2025.05.15.654154; this version posted May 19, 2025. The copyright holder for this preprint (which was not certified by peer review) is the author/funder, who has granted bioRxiv a license to display the preprint in perpetuity. It is made available under aCC-BY 4.0 International license.

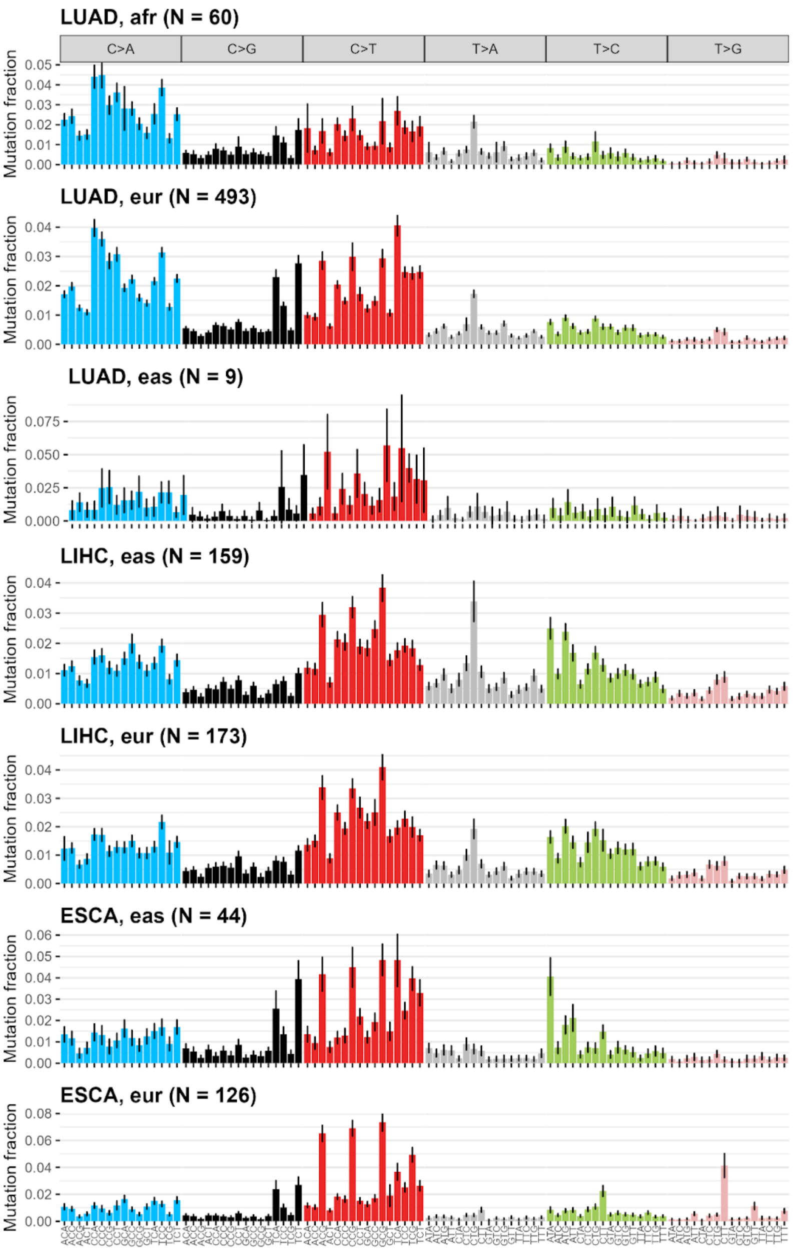

Supp Fig 8. Aggregate spectra for Lung adenocarcinoma (LUAD), liver hepatocellular carcinoma (LIHC), and esophageal carcinoma (ESCA) by ancestry. Bars denote spectra means (all samples weighted equally). Error bars denote 95% confidence intervals from the standard error of the mean ($\sigma / \sqrt{n}$). Notable differences include C>A in LUAD (SBS4), T>A and T>C in LIHC (SBS22 and SBS16, respectively), and T>C and T>G in ESCA (SBS16 and SB17b, respectively).

bioRxiv preprint doi: https://doi.org/10.1101/2025.05.15.654154; this version posted May 19, 2025. The copyright holder for this preprint (which was not certified by peer review) is the author/funder, who has granted bioRxiv a license to display the preprint in perpetuity. It is made available under aCC-BY 4.0 International license.

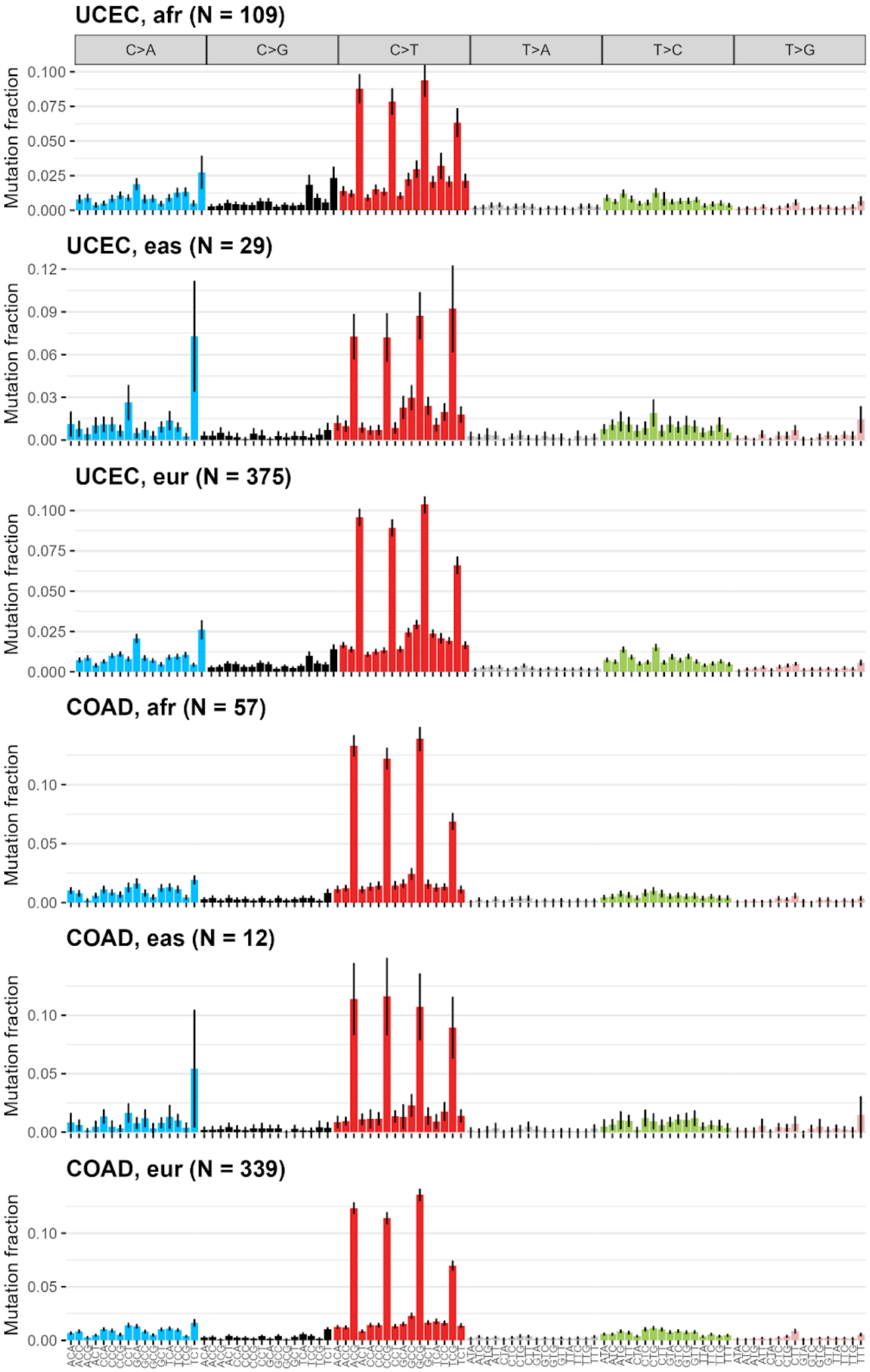

Supp Fig 9. Aggregate spectra results for Uterine (Uterine corpus endometrial carcinoma - UCEC) and colorectal adenocarcinoma (COAD) cancers. Bars denote spectra means (all samples weighted equally). Error bars denote 95% confidence intervals from the standard error of the mean ($\sigma / \sqrt{n}$). Notable differences include TCT>TAT (SBS10a) and TCG>TTG (SBS10b).
